# Character Strengths: Person–Environment Fit and Relationships With Job and Life Satisfaction

**DOI:** 10.3389/fpsyg.2020.01582

**Published:** 2020-07-23

**Authors:** Fabian Gander, Jennifer Hofmann, Willibald Ruch

**Affiliations:** Department of Psychology, LIVES – Overcoming Vulnerability: Life Course Perspectives, University of Zurich, Zurich, Switzerland

**Keywords:** job satisfaction, person job fit, life satisfaction, person–environment (P–E) fit, character strengths

## Abstract

Several studies demonstrated the relevance of character strengths in the workplace. For example, it has been shown that they positively relate to performance and are strong predictors of job satisfaction. Furthermore, it was demonstrated that occupational groups differ in their average levels of character strengths. However, little is known about the effects of the congruence between a person’s strengths profile with the average profile within an occupational group (environmental congruence) on well-being. In a nationally representative sample (*N* = 870) of employed adults, we analyzed data on character strengths (*t*1), and measures of job and life satisfaction at three different time points (*t*1–*t*3; separated by 1 year). We studied (1) whether employees in different occupational groups differ with regard to their levels and configurations of character strengths, (2) how levels and configurations of character strengths relate to concurrent and predictive job and life satisfaction, and (3) whether a fit between strengths of a person and the environment goes along with current and future job and life satisfaction. Results confirmed previous findings that small, but meaningful, differences in character strengths among employees in different occupational groups can be found and that character strengths positively relate to current and prospective job and life satisfaction. Furthermore, results suggested that a better person–environment fit goes along with higher job and life satisfaction. These results suggest character strengths and could play an important role in vocational and career counseling.

## Introduction

Character strengths are a set of 24 positively valued traits, as summarized in the Values in Action (VIA) classification (Peterson and Seligman, 2004). Several character strengths were found to positively relate to numerous desirable outcomes at work, such as work performance or satisfaction, and the occurrence of fewer undesirable outcomes, such as stress or counterproductive work behavior (e.g., Harzer and Ruch, 2014, 2015; Littman-Ovadia and Lavy, 2016; Heintz and Ruch, 2020). Specifically, higher expressions in character strengths make better and more satisfied employees, as summarized by Peterson et al. (2009); p. 229: “No matter the occupation, character matters in the workplace.” While many character strengths indeed are beneficial at work regardless of the occupation, the congruence between strengths of the person and those demanded by the environment might also play a role. When interested in finding the best job for a person—for example, the goal of vocational, and career counseling settings—one frequent approach is taking both the characteristics of the person and those of the environment into account and searching for an optimal fit between the two. Thus, in line with this notion, one would not only expect that character strengths in general are beneficial to workplace outcomes, but specific strengths of the person that are suited to certain workplaces are most beneficial. In line with this, a recent review by Van Vianen (2018) summarized the research on person–environment fit theory and concluded that best outcomes can be expected when the characteristics of the person and the environment are compatible. At the workplace, this conclusion is supported by findings on the fit of vocational interests to the workplace tasks (for reviews and meta-analyses, see Spokane et al., 2000; Kristof-Brown et al., 2005; Nye et al., 2017).

This idea of congruence is also one of the core tenets of Positive Psychology: Peterson and Seligman (2004) suggested that optimal outcomes are achieved when someone displays his or her highest character strengths on a regular basis. Applied to the workplace, this idea suggests that people should look for occupations in which their specific set of strengths (or at least some of them) is asked for and can be displayed. So far, there is only little research on the role of environmental congruence with regard to character strengths, even though it has been argued early on that character strengths might make an important addition to vocational and career counseling and might help in guiding people to occupations in which they are able to experience a high work and life satisfaction (Jungo et al., 2008).

In the present study, we study person–environment congruence with respect to character strengths on the level of occupational groups. We examine whether congruence between a person’s character strengths profile and the typical character strengths profile in his or her occupation goes along with higher job satisfaction and life satisfaction. Furthermore, we aimed at extending earlier research on the role of character strengths at the workplace in two regards. We study differences in character strengths among employees in different occupations using a comprehensive classification of occupations and a representative sample of the workforce. Finally, we examine the associations of character strengths with concurrent and predictive (i.e., assessed at a later time point) job and life satisfaction.

### Character Strengths in Different Occupations

There are several empirical hints toward differences among occupations with regard to the prototypical character strengths profile of the people working in these occupations. This was already shown by Peterson et al. (2009) who compared the average levels of character strengths among selected occupational groups, namely, managers, professionals, administrator, clerks, blue collar workers, and homemakers. They reported higher scores for professionals and managers in the strengths of creativity, curiosity, judgment, love of learning, perspective, perseverance, hope, and zest. Homemakers scored higher in kindness and love, while clerks and blue collar workers reported higher scores in humility. A recent study (Heintz and Ruch, 2020) compared the levels of character strengths across eight selected occupations (i.e., nurses, physicians, supervisors, clinical psychologists, office workers, social workers, and educators, economists, and teachers) and found group differences for all strengths except for kindness, self-regulation, and humor. These differences mostly followed the expected pattern; for example, social workers scored higher in teamwork than on average, psychologists in social intelligence, and supervisors and teachers in leadership. While in both studies the effects among different occupations were relatively small and some occupations (e.g., managers) scored highest in most strengths, the existence of such differences allows for comparing an individual’s profile across all 24 strengths with the typical profile within a given occupation. This comparison might be useful for career counseling or placement decisions.

So far, all studies compared selected occupations, and, to the best of our knowledge, no study has examined group differences in character strengths using a comprehensive classification such as the International Standard Classification of Occupations (ISCO; International Labour Office [ILO], 2012). We argue that this is relevant since it allows for considering all existing occupations in an internationally comparable framework. This study aims at closing this gap by investigating a representative sample of the Swiss workforce.

### Character Strengths and Job and Life Satisfaction

Various studies have been conducted on the relationships of character strengths with different indicators of well-being. Overall, findings suggest that almost all character strengths positively relate to subjective and psychological well-being (Hausler et al., 2017; Wagner et al., 2020), physical well-being (Proyer et al., 2013), general health (Gander et al., 2020), as well as life satisfaction (Buschor et al., 2013), and job satisfaction (Jungo et al., 2008; Heintz and Ruch, 2020). The exceptions are the strengths of modesty, prudence, appreciation of beauty and excellence, and judgment, for which often small negative relationships (modesty), no relationships, or small positive relationships with well-being (prudence, beauty, and judgment) are reported. For both job satisfaction and life satisfaction, usually the same set of five strengths (i.e., zest, hope, curiosity, love, and gratitude) yield the strongest relationships, while there were also some differences depending on the occupational group (Heintz and Ruch, 2020). While the existing studies focused on specific occupations, they also focused on concurrent relationships (i.e., assessed at the same time) of character strengths with well-being, but did not examine whether character strengths are also associated with future well-being. Thus, the current study aims at closing this gap by including three measurement time points for the assessment of job or life satisfaction.

### Person–Environment Fit

Person–environment fit theory suggests that “people have an innate need to fit their environments and to seek out environments that match their own characteristics” (Van Vianen, 2018; p. 77). Overall, congruence can be studied on the level of individuals, groups, occupations, or organizations. An important distinction has been made between supplementary congruence (i.e., an individual “supplements, embellishes, or possesses characteristics which are similar to other individuals”; Muchinsky and Monahan, 1987; p. 269), and complementary congruence (i.e., a “weakness or need of the environment is off-set by the strength of the individual, and vice versa”; Muchinsky and Monahan, 1987; p. 271). Thus, supplementary fit is given, when the individual and his or her environment is similar, while complementary fit describes situations in which an individual provides aspects to the environment that are currently not represented (but demanded). Van Vianen (2018) describes person–vocation fit (e.g., a person’s vocational interests match the vocational characteristics) and person–job fit (e.g., a person’s abilities match those demanded by the job) as examples for complementary fit, while person–supervisor, person–team, and person–organization fit (e.g., a person’s attributes or values match those of the supervisor, team, or organization) are examples for supplementary fit.

Overall, both types of fit were found to go along with positive individual outcomes at work (Kristof-Brown et al., 2005). Furthermore, while fit shows the strongest associations to attitudinal outcomes, such as work satisfaction, it is lesser associated with behavioral outcomes such as work performance or turnover intentions. In addition, the direction of misfit (e.g., whether a person’s abilities exceed or are inferior to those demanded by the job) and the level of fit (e.g., whether a person with high abilities is in a job demanding high abilities, or a person with low abilities is in a matching job) seem to be of lesser relevance, thus supporting central tenets of person–environment fit theories (Van Vianen, 2018). While these theories have also been criticized (e.g., Edwards, 2008), they represent nonetheless a crucial concept within organizational behavior research.

### Character Strengths and Person–Environment Fit

The fundamental proposition of person–environment fit theories goes along very well with basic theoretical assumptions of character strengths. Peterson and Seligman (2004) suggested that displaying one’s highest character strengths goes along with beneficial outcomes for the individual. Thus, individuals should report the highest well-being, if they are in an environment, which asks for and benefits from the individuals’ strengths (cf. complementary fit). The idea of examining character strength-based person–environment fit at the workplace emerged early in the field of character research. Peterson et al. (2009) investigated whether those character strengths that are more typical for a specific occupation show stronger relationships to job satisfaction (supplementary fit), or whether those strengths that are “rare” within an occupation yield stronger relationships to job satisfaction (complementary fit). In their analyses, they correlated the group means of a character strength with the correlation between this character strength and life satisfaction in the respective group (i.e., they analyzed whether the average level of a strength in a given group goes along with this strength’s association with life satisfaction in this group). The authors report small negative correlations between the level of character strengths and the relationships between the strengths and job satisfaction within each studied occupation. Peterson et al. (2009) interpreted these relationships as supporting the idea of complementary fit and contradicting the notion of supplementary fit. Yet, one important limitation of this study is that the inference leading to a conclusion of complementary fit does not necessarily hold true: Simply because a strength is uncommon in a given occupation does not necessarily mean that it is also important in this occupation. Furthermore, this study did not directly examine fit by examining the congruence of each individual to his or her occupation, but relied on indirect inferences, by looking at the relationships between group levels of strengths and their relationships with job satisfaction.

Several studies have been conducted on the applicability of character strengths at work (e.g., Harzer and Ruch, 2012, 2013; Lavy and Littman-Ovadia, 2017; Höge et al., 2020; Huber et al., 2020; Strecker et al., 2020). These studies examined whether and how many of one’s highest character strengths, the so-called *signature strengths*, can be applied by individuals in their occupation (i.e., whether these strengths are encouraged, perceived as useful and important, and are actually displayed). Harzer et al. (2017) argued that this could be considered an example of complementary fit. In general, a positive association between the number of character strengths that can be applied and various positive outcomes, including job satisfaction, has been confirmed repeatedly (e.g., Harzer and Ruch, 2012; Harzer et al., 2017; Lavy and Littman-Ovadia, 2017; Huber et al., 2020). Yet, in these studies, the need for a particular strength at the workplace is often confounded with the frequency of this strength being displayed at the workplace and being considered as useful for completing tasks. Thus, this conceptualization of fit might overestimate the importance of person–environment fit, since it not only covers the needs and demands of the workplace. Overall, existing research hints toward positive effects of person–environment fit with respect to character strengths and work-based outcomes, but more research is needed that disentangles the information that serves into the indicators of fit.

### The Present Study

The aims of the present study were threefold. First, we examined whether occupational groups differ with regard to their levels and configurations of character strengths: We assume that occupational groups differ with regard to what character strengths are demanded; for example, social occupations should require higher levels of strengths of humanity, while academic occupations might require higher levels of cognitive strengths.

Following the suggestion that “individuals strive toward fit” (Van Vianen, 2018; p. 81) we therefore assume that the average levels of character strengths in employees of different occupational groups reflect those differences in demand. For categorizing the occupational groups, we used the ISCO (International Labour Office [ILO], 2012) that distinguishes among 10 occupational groups: (1) managers; (2) professionals; (3) technicians and associate professionals; (4) clerical support workers; (5) service and sales workers; (6) skilled agricultural, forestry, and fishery workers; (7) craft and related trades workers; (8) plant and machine operators and assemblers; (9) elementary occupations; and (10) armed forces occupations (the latter two groups were not considered in the present study due to the small number of participants in these occupations). We conducted all analyses using the absolute scores of character strengths (“levels”). Additionally, for reducing the influence of possible response biases, we also analyzed ipsative scores of character strengths (i.e., *z*-standardized within the individual; “configurations”); when using ipsative scores, people do not differ in their levels across all strengths (i.e., whether someone scores higher in all strengths), but only with regard to the configurations of their character strengths (i.e., whether someone scores higher in one strength than in another strength). As an example for illustrating the difference between these two approaches, assuming we consider three self-reported characteristics A, B, and C (rated on a scale ranging from 1 and 5) of two individuals *X* and *Y*. These individuals should be compared regarding how well their profile across these three characteristics fits to an optimal profile for a specific occupation, which is A = 5, B = 4, and C = 3. Person *X* reported scores of 4, 5, and 3, and Person *Y* scores of 3, 2, and 1, for A, B, and C, respectively. Thus, when considering absolute scores, Person *X* fits better to this occupation (sum of deviations from optimal profile = 2) than Person *Y* (sum of deviations = 6). However, we also note that Person *X* reported higher scores across all three characteristics than Person *Y*. Thus, instead of absolute scores, we might also look at the rank order of the characteristics (as a simple version of ipsative scores). Thereby, we would see that while Person *Y* perfectly replicates the rank order of characteristics demanded by the job (i.e., A > B > C) and would therefore be an optimal fit for the job, this is not the case for Person *X* (B > A > C). Overall, the two approaches might lead to different conclusions. Ipsative scores have the advantage that they are less prone to response biases but the disadvantage that also potentially important information is lost—that Person *X* reported higher scores across all three characteristics than Person *Y* might also be an adequate evaluation of their characteristics. Therefore, we decided to report results on both approaches with the idea to make use of all the information contained in absolute scores while also comparing these findings with results on less biased ipsative scores.

We are extending earlier findings on group-level differences (Peterson et al., 2009; Heintz and Ruch, 2020) by using a nationally representative sample of the workforce, a comprehensive classification of occupations, and considering both differences in levels and configurations of character strengths. We expected, in accordance with earlier findings (Peterson et al., 2009), higher scores in cognitive strengths (i.e., creativity, curiosity, judgment, love of learning, and perspective), as well as perseverance, hope, and zest in managers and professionals, and higher scores in leadership in managers as compared to the average across all occupational groups (grand mean).

Hypothesis 1: Occupational groups differ with regard the (a) levels and (b) configurations of character strengths.
Hypothesis 2: Managers report higher absolute and ipsative scores in the strengths of (a) creativity, (b) curiosity, (c) judgment, (d) love of learning, (e) perspective, (f) perseverance, (g) hope, (h) zest, and (i) leadership than on average.
Hypothesis 3: Professionals report higher absolute and ipsative scores in the strengths of (a) creativity, (b) curiosity, (c) judgment, (d) love of learning, (e) perspective, (f) perseverance, (g) hope, and (h) zest than on average.

Second, we studied the relationships of levels and configurations of character strengths with concurrent and predictive job and life satisfaction (assessed three times, separated by 1 year each). We are extending previous findings (e.g., Heintz and Ruch, 2020) by also considering the predictive validity of character strengths for job and life satisfaction as well as considering a nationally representative work force of a country (as compared to investigating selected groups of occupations). In line with earlier findings, we expected positive correlations for most strengths, with the highest relationships for zest, curiosity, hope, gratitude, and love. Furthermore, we expected that similar relationships (but smaller in size) are obtained for the assessments of job and life satisfaction at later time points.

Hypothesis 4: The absolute scores of (a) zest, (b) curiosity, (c) hope, (d) gratitude, and (e) love positively relate to concurrent and predictive job and life satisfaction (i.e., measured 1 and 2 years later).
Hypothesis 5: The ipsative scores of (a) zest, (b) curiosity, (c) hope, (d) gratitude, and (e) love positively relate to concurrent and predictive job and life satisfaction (i.e., measured 1 and 2 years later).

Third, we were interested in whether there is an effect of environmental congruence with regard to character strengths on the level of occupational groups. Following the assumptions that differences among occupational groups in character strengths are meaningful representations of those character strengths demanded in those occupations, and that displaying one’s strengths is fulfilling, we expected positive relationships of environmental fit to well-being. We analyzed whether the convergence between an individual’s strengths profile with his or her occupational group’s strengths profile is related to job and life satisfaction, both concurrent and predictive. We studied both job and life satisfaction, since given that people spend a lot of time at work, we assumed that person–environment fit would not only affect job satisfaction but also life satisfaction. We are extending earlier findings (Peterson et al., 2009; Harzer et al., 2017) by estimating the degree of congruence between each participant and his or her occupational group with regard to levels and configurations of character strengths. Furthermore, we are extending previous studies by also considering predictive validity of environmental congruence on job and life satisfaction. In line with person–environment fit theory, studies on vocational interests (e.g., Kristof-Brown et al., 2005), and findings on aspects of complementary fit (Harzer et al., 2017), we expected that the better one’s character strengths profile converges with his or her occupational group, the higher levels of job and life satisfaction are reported.

Hypothesis 6: The absolute fit of an individual’s character strengths profile with the profile of the corresponding occupational group positively relates to concurrent and predictive job and life satisfaction (i.e., measured 1 and 2 years later).
Hypothesis 7: The ipsative fit of an individual’s character strengths profile with the profile of the corresponding occupational group positively relates to concurrent and predictive job and life satisfaction (i.e., measured 1 and 2 years later).

## Materials and Methods

### Sample

The data of *N* = 870 adults (51.6% women) aged between 27 and 57 (*M* = 44.35; *SD* = 8.27) at *t*1 was analyzed. All participants were working and living in Switzerland. The sample was representative of the Swiss workforce. A large part of the sample (37.9%) completed tertiary education (e.g., university), about half of the sample (52.1%) completed secondary education (e.g., vocational training), 3.7% completed primary education, while 6.3% had another educational background or did not indicate their educational level. Most ISCO groups (i.e., 8 out of 10) were represented in the sample: managers (11.5%); professionals (31.7%); technicians and associate professionals (20.9%); clerical support workers (10.2%); service and sales workers (10.2%); skilled agricultural, forestry, and fishery workers (3.0%); craft and related trades workers (8.5%); plant and machine operators, and assemblers (3.9%). Due to the small number of people in elementary occupations (eight participants), this occupational group was excluded from further analyses. In addition, there were no people working in armed forces occupations, in line with the expectations. Most participants (56.6%) worked full time, while overall, participants were working between 10 and 100% (*M* = 85.54%; *SD* = 19.96%; full-time equivalent).

Of those who completed *t*1, *n* = 690 completed *t*2 and *n* = 677 completed *t*3, while *n* = 587 (67.5%) completed both waves. Analyses of dropouts revealed no differences at *t*1 for gender [χ^2^(1, *N* = 870) = 0.00, *p* = 0.994], education [χ^2^(3, *N* = 870) = 5.51, *p* = 0.138], occupational group [χ^2^(7, *N* = 870) = 7.66, *p* = 0.364], nor job satisfaction [*t*(866) = 0.47, *p* = 0.638], or life satisfaction [*t*(868) = 0.14, *p* = 0.892]. However, those who missed at least one assessment were on average 1.62 years younger [*t*(868) = 2.70, *p* = 0.007] than those who completed all three assessments.

### Measures

The *Character Strengths Rating Form* (CSRF) is a 24-item self-report instrument for the assessment of the 24 character strengths of the VIA classification (Peterson and Seligman, 2004). It utilizes one short description for each of the strengths that is rated on a 9-point Likert-style scale (from 1 = “not like me at all” to 9 = “absolutely like me”). A sample item is “Curiosity (interest, novelty-seeking, and openness to experience): Curious people take an interest in all ongoing experience in daily life for its own sake and they are very interested in, and fascinated by, various topics and subjects. They like to explore and discover the world, they are seldom bored, and it’s easy for them to keep themselves busy.” Ruch et al. (2014) report good convergent validity with the standard instrument for assessing character strengths, the VIA Inventory of Strengths (VIA-IS; Peterson et al., 2005), and Gander et al. (2020) provided information on its stability and criterion validity when predicting external criteria such as life satisfaction, mental health problems, or general health.

The *Satisfaction with Life Scale* (SWLS; Diener et al., 1985; used in the German adaptation as used by Ruch et al., 2010) is a five-item self-report instrument for the assessment of life satisfaction. The SWLS uses a 7-point Likert-style scale (7 = “strongly agree” to 1 = “strongly disagree”). A sample item is “In most ways, my life is close to my ideal.” The SWLS has frequently been used in research and shows good psychometric properties (Pavot and Diener, 2008). Internal consistency in the present sample was high at all measurement time points (*α* = 0.89/0.90/0.92), and the ratings were stable across the 3 years (*t*1–*t*2: *r*_*tt*_ = 0.74; *t*2–*t*3: *r*_*tt*_ = 0.72; and *t*1–*t*3: *r*_*tt*_ = 0.68).

*Job satisfaction* was assessed with five self-report items developed for this study (Massoudi, 2009) based on an adaption of the Minnesota Satisfaction Questionnaire (Weiss et al., 1967). The items cover different aspects of job satisfaction, including satisfaction with supervisor and colleagues, job security, salary, and working conditions and use a 4-point Likert-style scale (1 = “not satisfied at all” to 4 = “very satisfied”). A sample item is “I am satisfied with my working conditions.” Internal consistency in the present sample was satisfactory (*α* = 0.70 at all measurement time points), and the scores were rather stable (*t*1–*t*2: *r*_*tt*_ = 0.61; *t*2–*t*3: *r*_*tt*_ = 0.59; and *t*1–*t*3: *r*_*tt*_ = 0.52).

### Procedure

Participants were part of a national longitudinal research project conducted during seven consecutive years (NCCR-LIVES project: Swiss National Center of Competence in Research LIVES—Overcoming vulnerability: Life course perspectives; Maggiori et al., 2016). Participants were randomly sampled based on information of the Swiss Federal Statistics Office. They completed the surveys on phone, paper, online, or a combination of phone/paper, phone/online. As an incentive for participation, all participants received gifts worth 20 Swiss Francs upon the completion of every year. In this article, we have used data from years 2 (=*t*1), 3 (=*t*2), and 4 (=*t*3) since character strengths were not assessed in the first project year. All participants provided informed consent for participation. No formal ethics approval was required for this study. All data used in this study are available upon request: https://forsbase.unil.ch/project/study-public-overview/14369/0/.

All analyses were controlled for influences of gender and age. We did not control for education, since the ISCO occupational groups are strongly related to education levels, and we consider the education level an important aspect of an occupation group, and not a confounding variable. In addition, from the perspective of vocational counseling, clients have often not reached their highest education level at the moment of the counseling, and thus, this information is not available at this point in time. Therefore, we conducted the main analyses without controlling for education, but additionally report a short summary of the findings when additionally controlling for education. A table of zero-order correlations among all studied variables is provided as an Online Supplementary.

## Results

### Differences in Strengths Among Occupational Groups

Means and standard deviations of character strengths in all eight occupational groups are provided as an online supplementary (see Online Supplementary Table A). In a multivariate analysis of covariance (MANCOVA), we compared the scores in all 24 character strengths (dependent variables) among the occupational groups (independent variable) while controlling for gender and age (covariates). Results suggested that the occupational groups differ with regard to the mean levels of character strengths, Pillai’s trace: *V* = 0.26, *F*(168, 5,901) = 1.36, *p* = 0.001, η*_*p*_*^2^ = 0.037, in line with the expectations and replicating earlier findings.

We repeated the same analysis with ipsative scores within the participants by computing within-person *z*-scores. That is, for all strengths, we subtracted a participant’s mean score across all 24 character strengths and divided the results by a participant’s standard deviation across all 24 character strengths. Thus, the resulting strengths profiles do not differ in the level among participants but only in the configuration (the mean across all strengths within a participant equals 0, and the standard deviation equals 1). Results of the MANCOVA using ipsative scores also suggested differences in the character strengths across the occupational groups, Pillai’s trace: *V* = 0.26, *F*(161, 5908) = 1.42, *p* < 0.001, η*_*p*_*^2^ = 0.038, in line with the expectations.

To investigate the quality of the mean level differences, we computed univariate ANCOVAs for each character strength separately (again, for absolute, and ipsative scores). To determine which occupational groups differed, we computed *post hoc* tests (using an alpha error level of *p* < 0.05), contrasting each occupational group with the grand mean (see Table 1).

**TABLE 1:** Analysis of covariance (ANCOVA) results for differences among occupational groups in means and ranks of character strengths, controlled for sex and age.

	**Absolute scores**				**Ipsative scores**			
	***F*(7, 859)**	***P***	**Partial η^2^**	**Contrast**	***F*(7, 859)**	***p***	**Partial η^2^**	**Contrast**
Creativity	2.95	0.005	0.023	2 > *M*	3.05	0.004	0.024	2 > *M* > 4
Curiosity	2.73	0.008	0.022	2 > *M*	2.80	0.007	0.022	2, 8 > *M* > 6
Judgment	4.51	0.000	0.035	1, 2 > *M*	4.71	0.000	0.037	1, 2, 3 > *M*
Love of learning	3.68	0.001	0.029	1, 2 > *M*	4.15	0.000	0.033	1, 2 > *M* > 6
Perspective	2.41	0.019	0.019	1 > *M* > 5	1.51	0.161	0.012	–
Bravery	2.45	0.017	0.020	1 > *M* > 2	2.78	0.007	0.022	*M* > 2
Perseverance	1.05	0.393	0.008	–	0.33	0.939	0.003	–
Honesty	1.97	0.057	0.016		0.70	0.670	0.006	–
Zest	1.84	0.076	0.015	*–*	1.71	0.104	0.014	
Love	1.77	0.089	0.014	–	0.95	0.466	0.008	–
Kindness	1.42	0.194	0.011	–	0.61	0.751	0.005	–
Social intelligence	3.31	0.002	0.026	1, 2 > *M*	1.52	0.157	0.012	
Teamwork	0.45	0.869	0.004	–	2.36	0.022	0.019	3 > *M*
Fairness	1.34	0.229	0.011	–	1.04	0.401	0.008	–
Leadership	2.53	0.014	0.020	1 > *M* > 3	1.46	0.180	0.012	
Forgiveness	1.45	0.181	0.012	–	0.76	0.619	0.006	
Humility	0.98	0.447	0.008	–	1.82	0.080	0.015	
Prudence	1.35	0.225	0.011	–	2.11	0.041	0.017	6 > *M* > 1
Self-regulation	1.29	0.254	0.010	–	1.54	0.151	0.012	
ABE	1.19	0.305	0.010	–	1.19	0.309	0.010	–
Gratitude	0.80	0.591	0.006	–	1.10	0.361	0.009	–
Hope	1.14	0.334	0.009	–	0.19	0.988	0.002	–
Humor	0.55	0.795	0.004	–	1.05	0.393	0.008	–
Spirituality	1.40	0.201	0.011	–	1.21	0.293	0.010	–

**N* = 870. ABE, Appreciation of Beauty and Excellence. Contrasts: 1, managers; 2, professionals; 3, technicians and associate professionals; 4, clerical support workers; 5, service and sales workers; 6, skilled agricultural, forestry, and fishery workers; 7, craft and related trades workers; and 8, plant and machine operators, and assemblers. *M* = average across all occupational groups. Example: “1 > *M* > 2” indicates that managers scored higher and professionals scored lower than the average across all occupational groups (*p* < 0.05).*

Table 1 shows that for the absolute scores, differences in eight character strengths were observed: managers, and/or professionals scored higher than the other groups in six strengths: the strengths of wisdom (i.e., creativity, curiosity, judgment, love of learning, and perspective), and social intelligence. Additionally, managers scored higher in leadership. Moreover, professionals, technicians, and service and sales workers scored lower than average in the strengths of bravery, leadership, and perspective, respectively.

Highly similar results were obtained for ipsative scores, although no group differences for perspective, social intelligence, and leadership were found. Yet, technicians and associate professionals and skilled agricultural, forestry, and fishery workers scored higher in team work and prudence, and managers scored lower in prudence as compared to the other groups, instead. However, again, managers, and/or professionals scored higher in the remaining strengths of wisdom, while professionals showed lower scores in bravery.

When conducting the same analyses while also controlling for education, no group differences could be observed in the MANCOVAs, Pillai’s trace: *V* = 0.193, *F*(168, 5,880) = 1.00, *p* = 0.506, and η*_*p*_*^2^ = 0.028 (absolute scores); Pillai’s trace: *V* = 0.207, *F*(161, 5,887) = 1.11, *p* = 0.157, and η*_*p*_*^2^ = 0.030 (ipsative scores). Univariate analyses (not shown in detail) suggested that no group differences in the strengths of wisdom were present when controlling for education, while the pattern for the other strengths remained unchanged, in line with the expectations.

### Relationships With Job and Life Satisfaction

For examining the relationships of character strengths with job and life satisfaction, we computed partial correlations between the character strengths at *t*1 with job and life satisfaction at *t*1, *t*2, and *t*3 while controlling for gender and age (see Table 2). Again, we repeated these analyses with absolute and ipsative scores.

**TABLE 2:** Partial correlations of character strengths (*t*1) with job satisfaction and life satisfaction (*t*1–*t*3), controlled for sex and age.

	**Absolute scores**						**Ipsative scores**					
	**Job satisfaction**			**Life satisfaction**			**Job satisfaction**			**Life satisfaction**		
	***t*1**	***t*2**	***t*3**	***t*1**	***t*2**	***t*3**	***t*1**	***t*2**	***t*3**	***t*1**	***t*2**	***t*3**
Creativity	0.11**	0.14***	0.07	0.16***	0.12***	0.13***	0.03	0.06	0.01	0.03	0.03	0.01
Curiosity	0.06	0.08*	0.04	0.17***	0.12***	0.13***	–0.02	0.01	–0.02	0.02	0.00	–0.02
Judgment	0.08*	0.06	0.07*	0.15***	0.10**	0.10**	–0.01	–0.04	0.00	–0.03	–0.06	−0.08*
Love of learning	0.07*	0.08*	0.06	0.17***	0.13***	0.16***	–0.02	0.01	0.00	0.00	0.01	0.01
Perspective	0.10**	0.09**	0.08*	0.20***	0.17***	0.16***	0.03	0.00	0.04	0.04	0.04	0.02
Bravery	0.09**	0.08*	0.01	0.17***	0.16***	0.14***	0.02	0.03	–0.04	0.00	0.04	–0.01
Perseverance	0.10**	0.08*	0.00	0.21***	0.11***	0.15***	0.02	0.00	−0.08*	0.05	–0.01	0.02
Honesty	0.10**	0.08*	0.05	0.16***	0.13***	0.10**	0.01	–0.01	–0.04	0.00	0.00	–0.06
Zest	0.16***	0.14***	0.10**	0.31***	0.27***	0.27***	0.08*	0.08*	0.06	0.16***	0.16***	0.14***
Love	0.15***	0.14***	0.13***	0.31***	0.26***	0.29***	0.05	0.04	0.06	0.17***	0.15***	0.15***
Kindness	0.16***	0.14***	0.11**	0.15***	0.10**	0.10**	0.04	0.02	0.01	–0.05	−0.08*	−0.07*
Social intelligence	0.12***	0.12***	0.15***	0.24***	0.20***	0.24***	0.02	0.03	0.11**	0.05	0.06	0.09**
Teamwork	0.09**	0.06	0.11**	0.13***	0.10**	0.13***	0.01	–0.05	0.05	−0.07*	–0.03	–0.03
Fairness	0.12***	0.06	0.10**	0.11***	0.09*	0.13***	0.00	–0.04	0.02	−0.09*	–0.06	–0.03
Leadership	0.14***	0.13***	0.08*	0.23***	0.16***	0.23***	0.08*	0.08*	0.04	0.10**	0.07	0.11***
Forgiveness	0.13***	0.12***	0.13***	0.14***	0.11***	0.16***	0.05	0.05	0.08*	–0.03	0.00	0.03
Humility	0.01	–0.03	0.01	0.00	–0.04	–0.02	−0.07*	−0.12***	−0.09*	−0.17***	−0.17***	−0.17***
Prudence	0.03	0.03	0.00	0.05	0.01	0.05	−0.09**	−0.07*	−0.11**	−0.14***	−0.15***	−0.13***
Self-regulation	0.07	0.05	0.06	0.14***	0.09*	0.15***	–0.03	–0.03	0.00	–0.02	–0.05	0.01
ABE	0.07*	0.07	0.08*	0.07	0.04	0.09**	–0.03	–0.01	0.04	−0.13***	−0.10**	–0.05
Gratitude	0.13***	0.11**	0.10**	0.23***	0.19***	0.21***	0.03	0.00	0.02	0.06	0.03	0.06
Hope	0.13***	0.13***	0.09*	0.34***	0.28***	0.29***	0.03	0.05	0.02	0.20***	0.18***	0.16***
Humor	0.11**	0.10**	0.07	0.20***	0.17***	0.18***	–0.02	–0.01	–0.02	0.02	0.04	0.03
Spirituality	–0.03	0.03	–0.02	0.08*	0.09**	0.07*	−0.10**	–0.03	−0.07*	−0.07*	–0.03	−0.07*

**N* = 868–870 (*t*1), *N* = 684–690 (*t*2), and *N* = 668–677 (t3). ABE, Appreciation of Beauty and Excellence. ^∗^*p* < 0.05, ^∗∗^*p* < 0.01, and ^∗∗^*p* < 0.001 (two-tailed tests).*

Table 2 shows that almost all character strengths (absolute scores) positively related to life satisfaction at all time points; exceptions were humility, prudence, and appreciation of beauty and excellence. Most character strengths also showed positive correlations to job satisfaction, with the most consistent relationships (i.e., present at all three time points) observed for hope, zest, love, kindness, gratitude, perspective, social intelligence, leadership, and forgiveness. Fewer relationships were obtained when using ipsative scores: The strengths of zest, love, and hope yielded consistent positive relationships, and the strengths of humility and prudence yielded consistent negative relationships with life satisfaction. For job satisfaction, consistent negative relationships were found for humility and prudence, equivalent to the findings for life satisfaction. These patterns remained widely unchanged when additionally controlling for education. Job and life satisfaction were moderately positively correlated, *r* = 0.34, *p* < 0.001.

### Relationships of Convergence Between Individual and Occupational Profile With Job and Life Satisfaction

Next, we were interested in the convergence of an individual’s strengths profile with the profile of his or her occupational group. For this purpose, we computed the Euclidian distance between a person’s strengths profile and the profile of his or her occupational group (i.e., the square root of the sum of the squared difference between every strength of the individual and his or her occupational group). The resulting fit index is a measure of dissimilarity, with higher scores denoting a lower fit of the person to the profile of his or her occupational group and lower numbers indicating a higher fit. Such fit indices were computed for both, absolute and ipsative scores in character strengths. Then, we computed partial correlations between these fit indices and job and life satisfaction, while controlling for gender and age (see Table 3).

**TABLE 3:** Partial correlations of job satisfaction and life satisfaction (*t*1–*t*3) with the fit of an individual’s character strengths profile with the profile of the corresponding occupational group (*t*1), controlled for sex and age.

	**Fit (absolute scores)**	**Fit (ipsative scores)**
**Job satisfaction**		
*t*1	0.03	−0.10**
*t*2	0.04	−0.03
*t*3	0.03	−0.08*
**Life satisfaction**		
*t*1	−0.11***	−0.09**
*t*2	−0.06	−0.07*
*t*3	−0.13***	−0.08*

**N* = 868–870 (*t*1), *N* = 684–690 (*t*2), and *N* = 668–677 (t3). Absolute fit = Euclidian distance between the character strengths raw scores of an individual and his/her occupational group’s scores. Relative fit: Euclidian distance between the character strengths ipsative scores of an individual and his/her occupational group’s ipsative scores. ^∗^*p* < 0.05, ^∗∗^*p* < 0.01, and ^∗∗^*p* < 0.001 (one-tailed tests).*

Table 3 shows that a better fit (lower scores in the fit indices) did go along with higher ratings of life satisfaction for both, absolute and ipsative scores (although not at *t*2 for absolute scores). Job satisfaction was negatively related to the fit indices when using ipsative scores (indicating that a better fit goes along with higher satisfaction) but showed no relationships when using absolute scores. The same pattern was obtained when additionally controlling for education. The fit indices for absolute and ipsative scores showed small positive correlations, *r* = 0.16, *p* < 0.001.

## Discussion

The present study examined the levels and configurations of character strengths with regard to differences between occupational groups, and the relationships to concurrent and predictive job and life satisfaction, and studied the relationships of person–environment fit (environmental congruence) in character strengths with concurrent and predictive job and life satisfaction.

Most importantly, our results showed higher levels of congruence between the character strengths of a person and those of the employees in his or her occupational group to go along with higher levels in current and future job and life satisfaction, providing evidence for effects of person–environment fit. Thus, our results were mostly in line with our expectations based on person–environment fit theory (e.g., Van Vianen, 2018) and earlier findings on effects of character strengths congruence (Harzer et al., 2017).

When looking at operationalizations of fit that have been used in past research, our findings disagree with some results by Peterson et al. (2009): They computed, for each occupational group separately, the average level of each character strength and the relationship of the character strength with work satisfaction within this group. Afterwards, they correlated the group means with the correlation between the character strengths and life satisfaction and found negative relationships (e.g., the higher the average level of a strength within a group, the lower the work satisfaction). While Peterson et al. (2009) conducted their analyses on the levels of occupational groups, we conducted our analyses based on the individuals, which has the advantage that it uses a larger data basis. When repeating the same analyses as Peterson et al. (2009); not shown in detail, we could not replicate their findings either: While they reported negative relationships between the (group) level of the character strength and its relationship to job satisfaction in all occupations, we found both positive and negative rank-order correlations job life satisfaction, depending on the occupation group [median *r*_*s*_(22) = 0.20, *p* = 0.349].

While for life satisfaction, the positive effect of congruence was found for both the levels and configurations of character strengths (supporting hypotheses 6 and 7 for life satisfaction), only effects for configurations were found for job satisfaction (supporting hypothesis 7 but not 6 for job satisfaction). Thus, discrepancies to the typical profile in an occupation that are only due to differences in levels seem to be of lesser relevance, while differences in configurations of strengths are relevant for both job satisfaction and life satisfaction. The latter finding is especially relevant, since it cannot be explained by response patterns such as acquiescence biases; while absolute scores of character strengths (levels) contain more information, ipsative scores (configurations) are less susceptible to such biases, and findings based on these scores are presumably more robust. Future research might more often consider studying both approaches—while generally stronger associations of strengths with outcomes can be expected when using absolute scores, some relevant findings might also be hidden by the differences in the levels of character strengths, especially when interested in character strengths profiles. For practical purposes, using ipsative scores might be especially interesting, for two main reasons: first, the reduction in response biases, which might be particularly relevant when strengths are also used in assessment situations, and second, clients can easily relate to the concept of a rank order and it breaks down the complexity of 24 strengths into a hierarchy that people can associate with.

Furthermore, the results showed positive relationships of most strengths with job satisfaction and life satisfaction that were widely in line with expectations and earlier findings (e.g., Ruch, 2008; Heintz and Ruch, 2020): When analyzing the levels of strengths, robust relationships were obtained for zest, love, gratitude, and hope, while curiosity was only related to life, but not job satisfaction (thus, supporting hypothesis 4 for all strengths except for curiosity). Instead, other strengths, such as perspective, kindness, social intelligence, leadership, and forgiveness showed positive relationships with both outcomes across all three time points. When looking at the configurations of strengths, consistent positive associations were found for zest, love, and hope for life satisfaction and consistent negative relationships for humility and prudence for both job and life satisfaction. Thus, hypothesis 5 was only supported for zest, partially supported for love and hope (with regard to life satisfaction), and not supported for curiosity and gratitude. Thus, for most strengths, the level of the strength seems to play a much more important role than the configuration. Only for zest, love, hope, humility, and prudence the relative standing within an individual also plays a role. Interestingly, the size of the correlations across the different time points only differed marginally; thus, character strengths seem to be also helpful for predicting future job and life satisfaction.

Finally, the results suggested differences in configurations and levels of occupational groups (in support of hypothesis 1). Again, these were mainly in line with our expectations and earlier research (Peterson et al., 2009): Managers scored higher in perspective and leadership, professionals scored higher in creativity and curiosity, and both scored higher in judgment and love of learning than on the average. The expectations with regard to other strengths (i.e., perseverance, hope, and zest) were not confirmed. Results were mostly parallel when looking at the configurations of strengths (with the exception of perspective, and leadership). Thus, hypothesis 2 (with regard to managers) was supported for judgment, love of learning, partially supported for perspective and leadership (only effect for absolute scores), and not supported for creativity, curiosity, perseverance, hope, and zest. Hypothesis 3 (with regard to professionals) was supported for creativity, curiosity, judgment, and love of learning, and not supported for perspective, perseverance, hope, and zest.

Interestingly, it was mostly professionals and managers who stood out from the remaining occupations with regard to character strengths, and it was mostly cognitive strengths that distinguished between these and other occupations. While the finding that cognitive strengths are higher in managers and professionals is not surprising since these occupations in general go along with a higher cognitive demand and higher educational requirements, it is interesting that no specific patterns for the other occupations were observed. One possible reason is the comparably smaller sample sizes in other occupations. However, one might also argue that the ISCO classification does not necessarily represent the psychological differences between occupations; for example, technicians and associate professionals represent a highly heterogeneous group covering occupations in the health, business, engineering, legal, and information technology domain; other categorizations, for example based on Holland’s (1997) typology, might be better suited for analyzing psychological differences.

Several further limitations have to be taken into account. First, most relationships were rather small by conventional standards. This can partially be explained by the use of a short form for the assessment of character strengths (i.e., the Character Strengths Rating Form; CSRF); studies that compared findings of this instrument with the standard instrument (i.e., the VIA-IS) confirmed that, when using the CSRF, relationships are generally underestimated but show a highly similar pattern (Ruch et al., 2014; Gander et al., 2020). Nonetheless, most participants scored rather high in most strengths, and more fine-grained measures of character strengths might yield more appropriate estimates. Second, despite the use of a large database, sample sizes for some occupations were rather small, while elementary and armed forces occupations were not represented. Thus, the reported findings are most representative for managers, professionals, and technicians and associate professionals while potentially less reliable for the remaining occupations. Fourth, the ISCO classification represents a categorization at the broadest level, and more proximal measures would certainly allow for a more precise estimation of person–environment fit (Spokane et al., 2000). Thus, future studies might include individual descriptions of one’s job for corroborating our findings. Fifth, the use of discrepancy measures does not allow for determining the direction of the discrepancy, and questions such as whether it is better to score higher than lower in comparison to one’s occupation group remain unanswered (see also Edwards, 2008). Especially with regard to character strengths, for which it has been suggested that there is no such thing as having “too much” of a strength (Peterson and Seligman, 2004), it seems at least debatable whether one assumption of person–environment fit theory—that the direction of misfit is of lesser importance—holds. The same goes for the assumption that the congruence on a high level (i.e., a highly creative person in a job requiring high levels of creativity) is equally beneficial as the congruence on lower levels. Further studies using more sophisticated techniques such as response surface analysis (Edwards and Parry, 1993) could help answering these questions. While these were not applicable in the present study due to the design (comparison of individuals with their occupational group), future studies using individuals’ descriptions of one’s job could apply such analytic approaches. Sixth, the present study is not able to distinguish selection effects from adaptation effects. A low level of congruence might be ameliorated by job crafting (Wrzesniewski and Dutton, 2001); employees might alter their tasks or their relationships with coworkers. Alternatively, in line with Peterson and Seligman’s (2004) assumption that character strengths are stable but still malleable, employees might also adapt themselves and become more typical for a given occupation over time. Both aspects might also have affected the results of the present study. Furthermore, as shown in previous research and the current study, character strengths differ in their relationships to job and life satisfaction. While one might argue that considering only those strengths that yielded the strongest relationships with well-being for the analysis of fit would suffice, we think that considering all 24 strengths is important and we assume that the importance of fit also generalizes to other work-related outcomes. One goal of the present article was to argue for the consideration of character strengths profiles and environmental congruence in research and practice. Analyzing the complete profile seems like a good starting point, and future studies might provide more information on what strengths should be considered for which outcomes.

Overall, the present study further corroborated the notion that character strengths theory can benefit from theories of person–environment fit theories. At the same time, there are several future avenues for research that should help to develop stronger theories (Edwards, 2008), including a more precise understanding of the conditions under which person–environment fit in character strengths is beneficial, the comparison of different aspects of person–environment fit (e.g., the fit to a supervisor, the team, the organization, the job, and the organization, etc.), or the effects of interventions or character strength-based vocational counseling on person–environment fit and well-being. At the same time, the present manuscript further corroborates some basic assumptions about character strengths, namely, that people in environments in which their strengths are demanded and can be displayed report higher well-being. While certainly more research is warranted, the results of the present study might be relevant for vocational and career counseling or placement decisions. Considering character strengths might help to guide people to occupations they fit best and in which they are able to experience higher levels of work and life satisfaction. Of course, more fine-grained information on the demanded levels of character strengths in different occupations would be needed for this purpose. In addition, while the present study further corroborates the notion that character strengths play an important role at work, future studies should also examine whether considering character strengths in addition to more traditional variables in career counseling (such as vocational interests, the big five personality traits, or general intelligence) indeed yields an incremental benefit in the prediction of relevant work outcomes.

## Conclusion

With above-mentioned limitations in mind, we tentatively conclude that (1) environmental congruence (i.e., fit between one’s character strengths and those typical in an occupational group) with regard to the configuration of strengths goes along with higher concurrent and predictive job and life satisfaction; (2) character strengths configurations and levels go along with present and future job and life satisfaction; (3) most consistent and robust associations are found for strengths such as zest, love, and hope; and (4) there are meaningful differences among occupational groups with regard to levels and configurations of character strengths; mostly cognitive strengths distinguish between managers, professionals, and other occupations.

## Data Availability Statement

All data used in this study are stored in a repository and available upon request: https://forsbase.unil.ch/project/study-public-overview/14369/0/.

## Ethics Statement

This study was carried out in accordance with the recommendations of the Swiss Psychological Association. All subjects gave written informed consent in accordance with the Declaration of Helsinki. According to the guidelines of the University of Zurich, no formal ethics approval was required for this study.

## Author Contributions

FG: conception and design of the work, data analysis, and drafting of the manuscript. FG, JH, and WR: interpretation of data analysis and final approval of the published version. JH and WR: critical revision of the manuscript. All authors contributed to the article and approved the submitted version.

## Conflict of Interest

The authors declare that the research was conducted in the absence of any commercial or financial relationships that could be construed as a potential conflict of interest.
