# Supplementary material for: Character Strengths: Person–Environment Fit and Relationships With Job and Life Satisfaction
Source: Front Psychol. 2020 Jul 23;11:1582. doi: 10.3389/fpsyg.2020.01582 (PMC7390898; doi:10.3389/fpsyg.2020.01582)
Supplement: Supplementary file 2 [file Table_1.DOCX]

Online Supplementary

Table A

Means and Standard Deviations in the 24 Character Strengths Across the Eight Occupational Groups.

|  | ISCO Occupational Group | | | | | | | | | | | | | | | | |
| --- | --- | --- | --- | --- | --- | --- | --- | --- | --- | --- | --- | --- | --- | --- | --- | --- | --- |
|  | 1 (*n* = 100) | | 2 (*n* = 276) | | 3 (*n* = 182) | | 4 (*n* = 89) | | 5 (*n* = 89) | | 6 (*n* = 26) | | 7 (*n* = 74) | | 8 (*n* = 34) | |  |
|  | *M* | *SD* | *M* | *SD* | *M* | *SD* | *M* | *SD* | *M* | *SD* | *M* | *SD* | *M* | *SD* | *M* | *SD* |  |
| Creativity | 6.49 | 1.77 | 6.68 | 1.62 | 6.02 | 1.95 | 6.17 | 1.72 | 6.34 | 1.84 | 6.08 | 1.92 | 6.41 | 1.80 | 6.03 | 2.04 |  |
| Curiosity | 7.00 | 1.63 | 7.15 | 1.42 | 6.66 | 1.66 | 6.84 | 1.48 | 6.64 | 1.69 | 6.46 | 1.56 | 6.73 | 1.72 | 7.09 | 1.51 |  |
| Judgment | 7.18 | 1.41 | 7.11 | 1.40 | 6.73 | 1.46 | 6.62 | 1.47 | 6.43 | 1.51 | 6.62 | 1.65 | 6.49 | 1.58 | 6.53 | 1.56 |  |
| Love of learning | 7.14 | 1.51 | 7.09 | 1.46 | 6.70 | 1.55 | 6.71 | 1.79 | 6.45 | 1.61 | 6.38 | 1.96 | 6.53 | 1.63 | 6.38 | 1.63 |  |
| Perspective | 6.93 | 1.33 | 6.68 | 1.44 | 6.43 | 1.50 | 6.61 | 1.69 | 6.21 | 1.59 | 6.65 | 1.62 | 6.49 | 1.58 | 6.15 | 1.73 |  |
| Bravery | 6.74 | 1.46 | 6.02 | 1.67 | 6.28 | 1.64 | 6.48 | 1.58 | 6.17 | 1.67 | 6.69 | 1.44 | 6.35 | 1.48 | 6.47 | 1.75 |  |
| Perseverance | 7.18 | 1.34 | 6.83 | 1.62 | 6.79 | 1.50 | 6.99 | 1.39 | 6.69 | 1.45 | 7.12 | 1.40 | 6.93 | 1.26 | 6.71 | 1.55 |  |
| Honesty | 7.61 | 1.18 | 7.49 | 1.39 | 7.20 | 1.46 | 7.70 | 1.04 | 7.21 | 1.43 | 7.38 | 1.68 | 7.30 | 1.39 | 7.21 | 1.20 |  |
| Zest | 6.85 | 1.42 | 6.38 | 1.58 | 6.27 | 1.51 | 6.67 | 1.51 | 6.40 | 1.42 | 6.69 | 1.89 | 6.57 | 1.51 | 6.71 | 1.55 |  |
| Love | 7.09 | 1.38 | 7.08 | 1.50 | 6.76 | 1.54 | 7.04 | 1.62 | 6.79 | 1.58 | 7.12 | 1.56 | 7.07 | 1.49 | 6.79 | 1.41 |  |
| Kindness | 7.38 | 1.18 | 7.35 | 1.24 | 7.08 | 1.32 | 7.48 | 1.12 | 7.22 | 1.41 | 7.42 | 1.24 | 7.18 | 1.35 | 7.09 | 1.06 |  |

Table A (continued)

|  | ISCO Occupational Group | | | | | | | | | | | | | | | | |
| --- | --- | --- | --- | --- | --- | --- | --- | --- | --- | --- | --- | --- | --- | --- | --- | --- | --- |
|  | 1 (*n* = 100) | | 2 (*n* = 276) | | 3 (*n* = 182) | | 4 (*n* = 89) | | 5 (*n* = 89) | | 6 (*n* = 26) | | 7 (*n* = 74) | | 8 (*n* = 34) | |  |
|  | *M* | *SD* | *M* | *SD* | *M* | *SD* | *M* | *SD* | *M* | *SD* | *M* | *SD* | *M* | *SD* | *M* | *SD* |  |
| Social intelligence | 7.38 | 1.25 | 7.20 | 1.46 | 6.79 | 1.38 | 7.01 | 1.30 | 6.79 | 1.47 | 6.96 | 1.28 | 6.82 | 1.46 | 6.68 | 1.22 |  |
| Teamwork | 6.86 | 1.30 | 6.74 | 1.57 | 6.75 | 1.53 | 6.87 | 1.38 | 6.85 | 1.43 | 6.73 | 1.59 | 6.64 | 1.71 | 7.06 | 1.18 |  |
| Fairness | 7.20 | 1.34 | 7.31 | 1.26 | 6.97 | 1.37 | 7.17 | 1.49 | 7.00 | 1.31 | 7.27 | 1.40 | 7.01 | 1.28 | 7.21 | 1.12 |  |
| Leadership | 7.05 | 1.48 | 6.45 | 1.72 | 6.16 | 1.73 | 6.36 | 1.69 | 6.21 | 1.88 | 6.69 | 1.93 | 6.54 | 1.62 | 6.68 | 1.41 |  |
| Forgiveness | 7.03 | 1.19 | 6.73 | 1.47 | 6.53 | 1.64 | 6.85 | 1.45 | 6.49 | 1.64 | 6.85 | 1.78 | 6.84 | 1.68 | 6.56 | 1.31 |  |
| Humility | 6.29 | 1.81 | 6.38 | 1.67 | 6.45 | 1.58 | 6.62 | 1.77 | 6.35 | 1.79 | 7.04 | 1.54 | 6.68 | 1.80 | 6.38 | 1.44 |  |
| Prudence | 6.32 | 1.65 | 6.43 | 1.57 | 6.41 | 1.67 | 6.66 | 1.55 | 6.35 | 1.61 | 7.27 | 1.04 | 6.49 | 1.63 | 6.44 | 1.38 |  |
| Self-regulation | 6.13 | 1.82 | 5.78 | 1.74 | 5.81 | 1.79 | 6.03 | 1.77 | 5.92 | 1.71 | 6.65 | 1.57 | 6.27 | 1.67 | 5.94 | 1.65 |  |
| ABE | 6.59 | 1.58 | 6.65 | 1.61 | 6.42 | 1.62 | 6.65 | 1.53 | 6.44 | 1.53 | 6.38 | 1.96 | 6.77 | 1.55 | 6.15 | 1.54 |  |
| Gratitude | 6.88 | 1.46 | 6.80 | 1.38 | 6.70 | 1.53 | 6.96 | 1.37 | 6.90 | 1.17 | 7.08 | 1.60 | 6.82 | 1.39 | 6.59 | 1.28 |  |
| Hope | 7.13 | 1.31 | 6.83 | 1.58 | 6.68 | 1.50 | 6.96 | 1.42 | 6.73 | 1.40 | 6.92 | 1.55 | 6.84 | 1.30 | 6.71 | 1.43 |  |
| Humor | 7.00 | 1.35 | 6.89 | 1.55 | 6.79 | 1.58 | 7.00 | 1.50 | 7.00 | 1.54 | 7.15 | 1.54 | 6.86 | 1.55 | 6.97 | 1.19 |  |
| Spirituality | 5.18 | 2.28 | 4.87 | 2.48 | 4.88 | 2.35 | 4.99 | 2.27 | 5.15 | 2.36 | 5.88 | 2.05 | 5.24 | 2.14 | 5.21 | 2.04 |  |

*Note*. *N* = 870. ABE = Appreciation of Beauty and Excellence. ISCO Occupational groups: 1 = Managers, 2 = Professionals, 3 = Technicians and associate professionals, 4 = Clerical support workers, 5 = Service and sales workers, 6 = Skilled agricultural, forestry and fishery workers, 7 = craft and related trades workers, 8 = plant and machine operators.
